# Therapeutic Potentials of Ketamine and Esketamine in Obsessive–Compulsive Disorder (OCD), Substance Use Disorders (SUD) and Eating Disorders (ED): A Review of the Current Literature

**DOI:** 10.3390/brainsci11070856

**Published:** 2021-06-27

**Authors:** Giovanni Martinotti, Stefania Chiappini, Mauro Pettorruso, Alessio Mosca, Andrea Miuli, Francesco Di Carlo, Giacomo D’Andrea, Roberta Collevecchio, Ilenia Di Muzio, Stefano L. Sensi, Massimo Di Giannantonio

**Affiliations:** 1 Department of Neurosciences, Imaging and Clinical Sciences, Università degli Studi G. D’Annunzio, 66100 Chieti-Pescara, Italy; giovanni.martinotti@gmail.com (G.M.); stefaniachiappini9@gmail.com (S.C.); alessio.mosca909@gmail.com (A.M.); andreamiuli@live.it (A.M.); francesco.dic@hotmail.it (F.D.C.); giacomo.dandrea1993@gmail.com (G.D.); rebeccacollevecchio@gmail.com (R.C.); dimuzioilenia@gmail.com (I.D.M.); stefano.sensi@unich.it (S.L.S.); digiannantonio@unich.it (M.D.G.); 2 Psychopharmacology, Drug Misuse and Novel Psychoactive Substances Research Unit, School of Life and Medical Sciences, University of Hertfordshire, Hertfordshire AL10 9AB, UK

**Keywords:** ketamine, esketamine, S-ketamine, obsessive–compulsive disorder (OCD), eating disorder, substance use disorder, anorexia, bulimia

## Abstract

The obsessive–compulsive spectrum refers to disorders drawn from several diagnostic categories that share core features related to obsessive–compulsive disorder (OCD), such as obsessive thoughts, compulsive behaviors and anxiety. Disorders that include these features can be grouped according to the focus of the symptoms, e.g., bodily preoccupation (i.e., eating disorders, ED) or impulse control (i.e., substance use disorders, SUD), and they exhibit intriguing similarities in phenomenology, etiology, pathophysiology, patient characteristics and clinical outcomes. The non-competitive N-methyl-D-aspartate receptor (NMDAr) antagonist ketamine has been indicated to produce remarkable results in patients with treatment-resistant depression, post-traumatic stress disorder and OCD in dozens of small studies accrued over the past decade, and it appears to be promising in the treatment of SUD and ED. However, despite many small studies, solid evidence for the benefits of its use in the treatment of OCD spectrum and addiction is still lacking. Thus, the aim of this perspective article is to examine the potential for ketamine and esketamine in treating OCD, ED and SUD, which all involve recurring and intrusive thoughts and generate associated compulsive behavior. A comprehensive and updated overview of the literature regarding the pharmacological mechanisms of action of both ketamine and esketamine, as well as their therapeutic advantages over current treatments, are provided in this paper. An electronic search was performed, including all papers published up to April 2021, using the following keywords (“ketamine” or “esketamine”) AND (“obsessive” OR “compulsive” OR “OCD” OR “SUD” OR “substance use disorder” OR “addiction” OR “craving” OR “eating” OR “anorexia”) NOT review NOT animal NOT “in vitro”, on the PubMed, Cochrane Library and Web of Science online databases. The review was conducted in accordance with preferred reporting items for systematic reviews and meta-analyses (PRISMA) guidelines. The use and efficacy of ketamine in SUD, ED and OCD is supported by glutamatergic neurotransmission dysregulation, which plays an important role in these conditions. Ketamine’s use is increasing, and preliminary data are optimistic. Further studies are needed in order to better clarify the many unknowns related to the use of both ketamine and esketamine in SUD, ED and OCD, and to understand their long-term effectiveness.

## 1. Introduction

Glutamatergic neurotransmission plays a critical role in modulating the activation of the cortico-striatal–thalamus circuitries that occur in OCD [1,2,3]. Several glutamate modulators, including memantine, topiramate and lamotrigine, have been evaluated, but they are still not approved for the treatment of OCD [1,3,4,5,6]. Ketamine, an aryl cyclohexylamine derivative synthesized in 1962, was first introduced into clinical practice as an intravenous (IV) anesthetic agent in 1970 [7]. Ketamine is currently prescribed for the induction and maintenance of general anesthesia via intramuscular (IM) or IV infusion; moreover, over the past two decades, it has emerged as a promising treatment for major depressive disorder (MDD) [8,9]. In addition, increasing evidence now points to its therapeutic efficacy in treatment-resistant depression (TRD), as well as a potential agent for reducing and preventing suicide in depressed patients, as it exerts rapid antidepressant properties as early as several hours after administration. This contrasts with traditional antidepressants (e.g., selective serotonin reuptake inhibitors [SSRIs]), that usually require several weeks for clinical responses [9,10,11,12,13,14,15]. An intranasal formulation of esketamine, currently branded as Spravato^®^, was approved by the Food and Drug Administration (FDA) in the United States in March 2019, and by the European Commission in December 2019, for [16,17] the treatment of TRD, defined as the lack of response to two or more adequate trials of antidepressant medications in a current moderate to severe depressive episode [12,17,18]. More recently, ketamine has been studied and used for several new indications, ranging from chronic pain to drug addiction and post-traumatic stress disorder [18,19,20]. The interest in the use of ketamine in OCD stems from its rapid antidepressant effect after only a single intravenous infusion [21] The rapid reduction in depressive symptom scores, even in those with refractory depression, led us to investigate its alleged use in OCD and OCD spectrum disorders.

### 1.1. Ketamine’s Mechanisms of Action

Ketamine is a racemic mixture composed of both R and S enantiomers (50% each). Compared to R-ketamine, esketamine (the S-enantiomer of racemic ketamine) is a more potent antagonist at the phencyclidine site of the ionotropic glutamate receptor, N-methyl-D-aspartate (NMDA), and it therefore offers a stronger analgesic potency than either R-ketamine or the racemic mixture [9]. Ketamine, through its action as an NMDA receptor (NMDAr) antagonist, produces a transient increase in glutamate release, while also activating the α-amino-3-hydroxy-5-methyl-4-isoxazole propionic acid receptor (AMPAr), which are both suggested to play a role in the antidepressant effects of ketamine [20,21]. It ultimately induces an array of molecular and cellular events [12,22,23] that include: (i) increases in brain-derived neurotrophic factor (BDNF) expression, synthesis and release [12,22,24]; (ii) activations of neurotrophic signaling pathways, such as extracellular signal-regulated kinase/mitogen-activated protein kinase (ERK/MAPK) and protein kinase B (AKT) [12,23]; (iii) inhibition of glycogen synthase kinase (GSK)-3; and (iv) activation of synaptic plasticity genes, such as those related to the activity-regulated cytoskeleton (ARC)-associated protein [20,25]. All these changes increase the levels of synaptic proteins and synaptogenesis, and eventually favor the restoration of synaptic function [9,12,23,25]; additional effects mediated by the modulation of monoaminergic neurotransmission cannot be excluded [12,22,23]. In this respect, one noteworthy observation is that acute and prolonged increases in dopamine levels in the prefrontal cortex, striatum and nucleus accumbens occur immediately after administering sub-anesthetic doses of esketamine [14]. Other possible cellular targets of ketamine include opioid (mu, delta and kappa) receptors, and ketamine binding to these receptors has been proposed to contribute to its anti-suicidal and antidepressant effects [16]. A better understanding of the impact of ketamine binding on serotonin, opioid, dopamine and cholinergic receptors [22,26] is needed for a full assessment of the therapeutic potentials of ketamine, especially with regard to mental health disorders. Of note, different preclinical models reported that it is unlikely that NMDA inhibition alone could play a major role in the antidepressant effects of (R,S)-ketamine, opening the door for future studies and major developments [27].

### 1.2. The Role of the Glutamatergic System in Mental Diseases and OCD

The excitatory neurotransmitter glutamate is prevalent throughout the central nervous system (CNS), and it has been found to play important physiological roles in neurodevelopment, cognition, learning and memory formation, as well as in the modulation of brain plasticity [28]. Glutamatergic neurons constitute approximately 80% of the synapses in the neocortex [29]. Glutamate activates various downstream pathways of nuclear genes by binding to a variety of membrane-bound receptors present on the postsynaptic membrane, which regulate secondary messenger systems. Excessive glutamate release can be toxic to the brain and has been linked to many neurodegenerative diseases, such as Alzheimer’s disease, amyotrophic lateral sclerosis and Huntington’s disease. A growing body of evidence also indicates that abnormalities in glutamatergic neurotransmission play an important role in the development of many major psychiatric disorders such as schizophrenia, bipolar disorder, major depressive disorder and OCD. Glutamatergic neurotransmission plays a critical role in modulating the activation of the cortico-striatal–thalamus circuitries that occur in OCD [1,2,3]. Several glutamate modulators, including memantine, topiramate and lamotrigine, have been evaluated, but they are still not approved for the treatment of OCD [1,3,4,5,6]. Recent preclinical, imaging and genetic studies support a specific role for glutamatergic transmission in OCD, also opening possible perspectives in the obsessive–compulsive spectrum [6]. The obsessive–compulsive spectrum refers to several mental health disorders encompassing several diagnostic categories that share core obsessive–compulsive features, such as anxiety, obsessive thoughts and compulsive behaviors [1]. Disorders that include these features can be grouped according to the focus of the symptoms, e.g., bodily preoccupation (i.e., eating disorders [ED] and body dysmorphic disorder) or impulse control (i.e., substance use disorder [SUD], gambling disorders, trichotillomania and other behavioral addictions). Although distinct from one another, these disorders share similarities in phenomenology, etiology, pathophysiology, patient characteristics and clinical outcomes. They can also be viewed as part of a continuum that spans from compulsivity to impulsivity, and they are characterized by harm avoidance as far as compulsion or risk-seeking for impulsivity. They also share a common neurobiological background, including the presence of abnormalities in the glutamatergic neurotransmission, that can be the target of common therapeutic interventions [30,31,32,33,34,35,36,37,38]. In this respect, Ketamine, which is able to produce a transient increase in glutamate release, could represent a valuable therapeutic option.

Aim of the study: To the authors’ knowledge, no review has synthesized the published data on the therapeutic potentials of ketamine and esketamine in the OCD spectrum by focusing on OCD, SUDs and EDs. In this perspective article, we have analyzed and reviewed current evidence on the drug efficacy of ketamine and esketamine in these mental disorders, and we have evaluated their advantages compared with currently available therapeutic tools. We have also outlined potential therapeutic opportunities for further investigation.

## 2. Methods

### 2.1. Data Extraction

The review was conducted in accordance with preferred reporting items for systematic reviews and meta-analyses (PRISMA) guidelines [39]. A literature search was performed using PubMed, Medline and Web-of-Science databases in April 2021, without any time restrictions. We used the following search terms: “ketamine” or “esketamine”) AND (“obsessive” OR “compulsive” OR “OCD” OR “SUD” OR “substance use disorder” OR “addiction” OR “craving” OR “eating” OR “anorexia”) NOT review NOT animal NOT “in vitro”. In addition, we performed further secondary searches by using the reference listing of all eligible papers.

### 2.2. Data Synthesis Strategy

**Figure 1:**
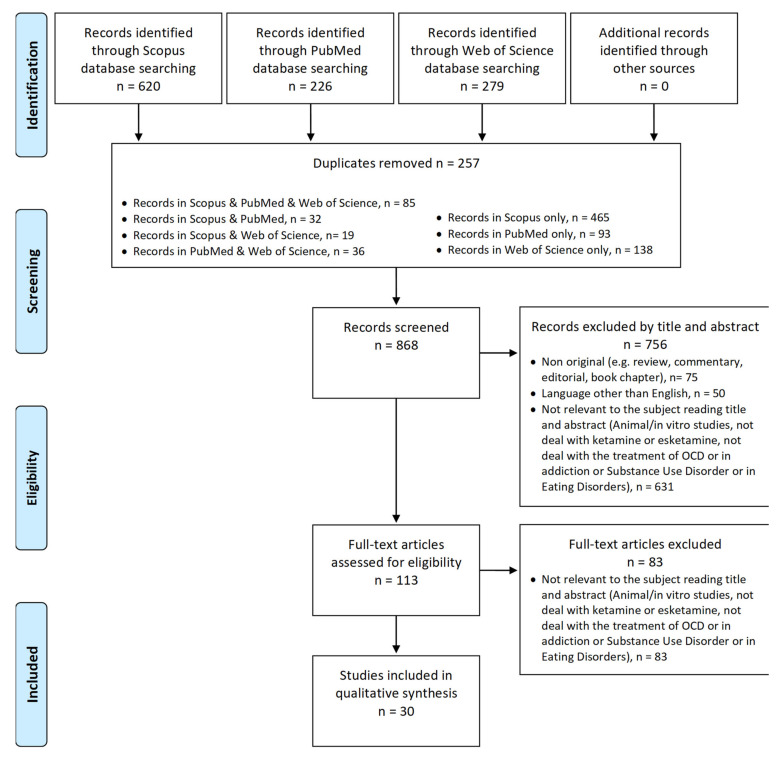
Flow-chart of study search and selection process according to PRISMA guidelines.

The search of results was carried out individually by five investigators (A.M. (Alessio Mosca), A.M. (Andrea Miuli), F.D.C., G.D., R.C., I.D.M.) and supervised by S.C. and M.P.; doubtful cases were discussed by G.M., M.D.G. and SS. The selection and eligibility phases of the articles were carried out independently by the five selected members and, afterwards, the papers were subjected to a final cross-check. Any questions not solved by the team related to understanding the topic covered in the articles were requested directly from authors, if they were contactable. The data were collected in a Word table containing first names and year of publication of the study, the design, demographic variables (gender, age, psychiatric history), details of the NPS taken (dosage, route of administration) and any other substances in combination, as well as the drugs’ effects on suicidal behaviors, suicidal ideation or abuse. Data synthesis was carried out by three independent members of the team (A.M. (Alessio Mosca), F.D.C. and G.D.) and compared at the end of the extraction process. All titles/abstracts were examined, and full texts of potentially relevant papers were obtained. Relevant works were selected in order to obtain a full representation of the available literature data on the selected topic. Experimental and observational studies, post-marketing surveillance reports, case reports, case series and fatality reports were included. The exclusion criteria included non-original research (e.g., reviews, commentaries, editorials, book chapters and letters to the editor), non-full-text articles (e.g., meeting abstracts), works in a language other than English and studies involving animal/in vitro experiments. Although letters to the editor, conference proceedings and book chapters were excluded from the literature review, they were still considered in the retrieval of further secondary searches. A.M. (Alessio Mosca), A.M. (Andrea Miuli), F.D.C., G.D., R.C. and I.D.M. independently extracted and collected relevant data; S.C., M.P., G.M., S.L.S. and M.D.G. contributed to the analysis of the results and discussed possible issues and disagreements during the revision of the paper. From an initial list of 1125 studies, duplicates (n = 257) were eliminated, as were papers unrelated to the topic (n = 631) and those that did not meet the inclusion criteria (e.g., reviews, letters to the editor, commentary, book chapter n = 75; non-English papers n = 50). A total of n = 113 full-text articles were assessed for eligibility. From them, a number of 83 full-text articles were excluded, as they were considered irrelevant to the subject when reading their titles and abstracts (animal/in vitro studies, not dealing with ketamine or esketamine, not dealing with the treatment of OCD, addiction, SUD or ED). Finally, 30 studies were included in the qualitative synthesis (Figure 1).

## 3. Results

A total of 30 eligible articles were finally identified and included in this systematic review. The findings are described in detail and organized in relation to the specific type of article and order of publication (see Table 1).

Studies retrieved were represented by nine case series/case reports [13,40,41,42,43,44,45,46,47,48], six randomized-controlled trials [49,50,51,52,53,54], ten open-label studies [55,56,57,58,59,60,61,62,63,64,65] and three double blind trials [66,67,68]. Most cases involved adult males (total M/F = 328/160= 2.1). Patients were mostly diagnosed with OCD [13,40,45,47,55,56,60,61,62,63,64] or a SUD, such as opioid dependence [41,43,44,45,46,57,58,59], cocaine dependence [49,50,52,66,67,68] or alcohol dependence [51,54,65]. Only three studies focused on ED [42,46,48]. Ketamine was mostly intravenously administered [42,43,44,45,47,48,49,50,51,52,53,54,55,56,57,59,60,61,65,66,67], but intranasal [40,62], intramuscular [46,58,68] and oral [13,41] routes of administration were also recorded. Ketamine was administered together with Cognitive Behavioral Therapy (CBT) [40,43], or in the context of ketamine-assisted psychotherapy (KPT) sessions [58,68] or Trauma Interventions using Mindfulness Based Extinction and Reconsolidation of memories (TIMBER) [59]. Ketamine was generally well tolerated, with the exception of transient effects, including nausea [42,43], headache [42,46], dry mouth [45], sleepiness [46], feelings of unreality [45], dissociation [46,49,55,56,62], dizziness [13], hallucinations [40,42,43], gaps in memory [55,56], sensory distortions [55,56], anxiety [43] and increases in systolic blood pressure [55,56,58]. Interestingly, unexpected suicidal thinking was observed, together with delayed-onset dysphoria and anxiety, in some patients diagnosed with OCD, but these patients were not recorded as being depressed [55,56].

### 3.1. OCD

According to the literature [3,40,55], ketamine via IV infusion significantly improves obsessive–compulsive symptomatology, with effects that are rapid (occurring in hours to minutes) but short-lasting (days to weeks) [13,40,45,47,55,56,60,61,62,63]. Interestingly, CBT prolongs ketamine effects [30,40,43,56,69]. Recently, a maintenance treatment with oral S-ketamine combined with experimental deep-brain stimulation showed long-term (18 months) beneficial effects in a patient with severe TRD and comorbid psychotic and obsessive–compulsive symptoms [13]. Two other studies showed that ketamine infusions were effective in patients diagnosed with treatment-resistant OCD, and that the ketamine effects on OCD symptoms, in contrast to depressive symptoms, did not seem to persist or progress after the acute effects of ketamine had dissipated [60,61]. A further proof-of-concept study that examined the time course of neurochemical effects of a single IV infusion of ketamine in OCD subjects confirmed that ketamine significantly increased gamma-aminobutyric acid (GABA) levels—but not glutamate levels—over time in the medial prefrontal cortex (MPFC). This increase was positively correlated with changes in OCD symptoms and was consistent with other recent studies suggesting that ketamine concurrently activates a subpopulation of GABAergic interneurons and projection neurons in the cortex [63].

### 3.2. SUD

A single-case trial with repeated ketamine IV infusions (0.5 mg/kg) over the course of 6 weeks produced positive results in a TRD patient with a co-occurring SUD (alcohol and benzodiazepine dependence) [65]. KPT was shown to reduce relapses in recently detoxified inpatients suffering from alcohol use disorder (AUD) [58,68,70]. Similarly, Dakwar et al. [50] evaluated the use of ketamine and motivational enhancement therapy in alcohol-addicted subjects and found positive effects in initiating and sustaining abstinence after 12 weeks of treatment. When combined with motivational enhancement therapy, the psychoactive/mystical effects of ketamine on alcohol-prone behavior were critical for producing a significant reduction in alcohol consumption [54]. Ketamine was also useful as adjunctive medication for managing severe alcohol withdrawal syndrome and reducing benzodiazepine requirements [71]. Interestingly, drinkers who are at risk of abuse benefited from ketamine infusions, in conjunction with a behavioral procedure called retrieval/destabilization focused on maladaptive alcohol reward memories; they showed reductions in the reinforcing behavior driven by drinking and decreased alcohol consumption when evaluated at the 9-month follow-up [53]. Currently, a phase II, multi-site, randomized, double-blind, placebo-controlled, parallel-group clinical trial is ongoing to investigate the effectiveness of IV ketamine in reducing relapse rates in recently detoxified alcohol-dependent individuals with depressive symptoms at the 6-month mark [72]. However, a recent study produced conflicting results on the combined use of naltrexone and ketamine for the treatment of patients with AUD and comorbid depression [63].

In Cocaine Use Disorder (CUD), a comparison with lorazepam infusion revealed that IV ketamine effectively enhanced the motivation to quit and reduced cocaine cravings. Remarkably, these results were obtained after the first ketamine infusion [66]. A comparison with the active control midazolam also showed significantly greater reductions in cocaine use, relapse rate and cravings with infusions of ketamine [50,52].

An evaluation of ketamine treatment in a 2-year follow-up study, including 70 heroin-dependent patients with Opioid Use Disorder (OUD) and randomized to a single low dose IM ketamine (0.2 mg/kg) injection or a high dose one (2 mg/kg), showed that KPT was effective at maintaining abstinence and reducing cravings within the first two years of follow-up. A greater and longer-lasting reduction in cravings was obtained with high doses rather than low doses of ketamine, indicating a dose dependence of the effects [58]. Moreover, multiple sessions of KPT resulted in significantly higher rates of 1-year abstinence and lower cravings [58,68]. A combination of ketamine, repetitive transcranial magnetic stimulation (rTMS) and mindfulness therapy appeared effective for maintenance of abstinence in OUD, particularly in terms of reducing cravings [56]. Ketamine was also found to be useful in suppressing the physiological symptoms of opioid withdrawal (e.g., increases in heart rate, cortisol levels and arterial pressure) [57,70].

### 3.3. ED

The first attempt to treat non-responder AN subjects with ketamine infusions was made in the late 1990s by Mills et al., who reported prolonged symptom remissions in 15 patients with a chronic, complex history of AN (i.e., comorbid compulsive eating [n = 4] and/or bulimia [n = 5]; 30% of the patients were a restrictive subtype of AN) after treatment with 2–9 ketamine infusions at intervals of 5–21 days [42]. IM ketamine was administered with repeat dosing at 4–6 week intervals in four patients chronically ill with an ED for more than 7 years, and current criteria for TRD show clinically meaningful changes in depression, and to a lesser degree anxiety, and ED symptoms [46]. An interesting recent case report of a 29-year-old woman with a long history of severe AN and intermittent alcohol dependence, and diagnosed with major depressive disorder, indicated complete recovery after a ketogenic diet associated with ketamine infusions [48].

## 4. Discussion

To the very best of our understanding, current data represent the first literature review focusing on the use of ketamine and esketamine as a treatment for OCD, SUDs and EDs. Overall, studies have been recorded in the past ten years, a signal that increased interest in the use of ketamine for the treatment of depression, as well as psychiatric disorders, has developed among researchers. Regarding current findings on ketamine as a therapeutic tool in OCD, SUD and ED, some considerations are needed.

### 4.1. Ketamine in OCD

The anti-obsessional effects of ketamine and NMDA receptor antagonists do show therapeutic potential. However, studies appear to be heterogeneous, and comparative studies with conventional therapeutic strategies were not reported in the current literature. The current evidence is still not strong enough to recommend ketamine for clinical practice at this time. The acute benefit of ketamine on OCD symptoms needs to be confirmed in controlled trials and cannot be divorced from the psychomimetic and dissociative effects of ketamine. Additionally, we cannot eliminate the possibility that other environmental effects caused acute changes in OCD symptoms. Current speculations are that the clinical effects may relate to the ketamine-induced mystical experiences rather than to a direct effect on NMDA receptors causing a transient increase in glutamate release [48,66]. A separate question is whether abnormalities in the glutamate system could be considered a fundamental cause of OCD, or whether they are a downstream consequence of other processes that are more primary to the pathophysiology. It is widely accepted that OCD is characterized by neuronal hyperactivity in definite key brain areas—mainly the orbitofrontal cortex, the caudate nucleus and the anterior cingulate cortex [73,74]. It is possible that the observed abnormalities in glutamate in patients with OCD are simply a consequence of this hyperactivity. In light of this, the role of ketamine/esketamine should be reconsidered. Future research is warranted to establish the parameters of ketamine’s efficacy. With larger clinical RCTs, a stronger argument can be made for future modifications of guidelines regarding patient care in this population.

### 4.2. Ketamine in SUD

Chronic use of addictive drugs produces enduring neuroadaptations in the corticostriatal glutamatergic brain circuitry. Increased susceptibility to relapse in drug-experienced individuals, even after long periods of withdrawal, might be associated with drug-related long-term morphological and electrophysiological changes in the glutamatergic synapses in the nucleus accumbens which, together with drug-induced transient morphological and electrophysiological changes, might both impair normal information processing and contribute to the uncontrollable motivation to relapse [75]. In the area of SUD, the use of ketamine appears to be promising, with most evidence in alcohol use disorder. This specific affect can be justified by the strongest role of glutamatergic neurotransmission in alcohol use disorders. The use and efficacy of ketamine in SUD are supported by the glutamatergic dysregulation and altered functioning of the prefrontal cortex and mesolimbic regions that occur in this condition [68,76,77,78]. Meye et al. [76] found that, in mouse lateral habenula (LHb) neurons targeting the rostromedial tegmental nucleus, cocaine enhanced glutamatergic transmission, increased excitability, influenced drug-driven aversive behaviors and shaped negative symptoms after drug taking. While a direct effect of ketamine on the reward system cannot be ruled out, a modulation of the reward pathway has been hypothesized [79,80]. In this regard, Williams at al. [81] reported that a single 50 mg dose of naltrexone dramatically blocked the rapid antidepressant and anti-suicidal effects of IV ketamine, while also reducing the rate of clinical response to ketamine from 71% to 0%. These findings suggest that opioid receptor activation plays a significant role in ketamine’s efficacy [81]. At odds with these findings, Yoon et al. [65] indicated that naltrexone pre-treatment did not interfere with the antidepressant effects of ketamine and that it enhanced the treatment of current major depressive disorder and comorbid AUD. In light of this, the promising results of ketamine should be interpreted with caution. If, on the one hand, ketamine’s efficacy could be mediated by the pro-glutamatergic effect, then, on the other hand, it may directly influence the opioid system and the reward chain. In this respect, ketamine may simply act as a modulator of the reward system.

### 4.3. Ketamine in ED

The number and quality of studies regarding ketamine and ED is still in its infancy, although glutamatergic neurotransmission plays an important role in ED, particularly in Anorexia Nervosa (AN) [82,83]. After encouraging results with some NMDA modulators, such as the partial agonist D-cycloserine [84,85] and the NMDA-r antagonist amantadine [86], ketamine has been tested in ED, with the rationale of normalizing glutamatergic transmission. At present, the data regarding the use of ketamine and esketamine in subjects diagnosed with an ED are preliminary, limited and related to AN only. Moreover, despite positive results in animal models of AN [87,88,89] in terms of food intake, weight gain and reduced hyperactivity, evidence is lacking regarding the effects of ketamine in AN patients. Nevertheless, more compelling and substantial evidence is needed from large cohort studies.

### 4.4. Possible Advantages of Ketamine and Esketamine Compared with Current Treatments

Treatment strategies for OCD, ED and SUD are complex and difficult, and they often involve pharmacological and psychological interventions. To date, several drugs have been approved for the treatment of patients affected by symptoms belonging to the OCD spectrum, ED and SUD [30,31,32,33,34,35,36,37,90,91]. However, in severe cases, in cases with dual diagnoses or in other vulnerable categories of patients, these therapies have often failed. In some cases, off-label use of serotonin and noradrenaline reuptake inhibitor (SNRI) antidepressants have produced beneficial effects in patients with OCD [30]. Off-label use of olanzapine also has shown promising results in ED [90,91,92], while the anticonvulsants gabapentin and pregabalin [93], and the antipsychotic aripiprazole [94], have shown some efficacy in increasing abstinence rates and managing alcohol withdrawal. However, rates of treatment resistance are high and innovative treatment options are warranted. In this respect, the use of new therapeutic pharmacological approaches, including psychedelic substances such as ketamine, to treat mental disorders is increasing [95], starting from the first assumptions made in the 1970s [96]. Preclinical studies have provided a significant scientific rationale for the potential of glutamate modulators in the management of anxiety disorders [1,60]. Indeed, using animal stress models, glutaminergic dysfunction in the prefrontal cortex and other limbic regions has been detected in both acute (shown to increase glutaminergic transmission) and chronic stress (associated with a decrease in glutamate receptors, resulting in lower glutamate transmission). These models have been used to assess the anxiolytic activity of drugs acting on NMDA, AMPA, kainite and mGLuR receptors [97]. Despite the fact that results are still preliminary, and mechanisms are incompletely understood, OCD-like behavior appeared to be sensitive to low doses of ketamine [98]. In this context, ketamine and esketamine are innovative treatments that generate a variety of largely unexplored and novel neuromodulatory activities. The preliminary evidence for the benefits of ketamine and esketamine use in these mental disorders is summarized in Table 2. 

As mentioned before [26], the improved prefrontal cortex glutamate homeostasis, increased synaptic functioning and enhanced BDNF signaling indicate that esketamine has the potential to be repurposed or further investigated as a treatment for OCD, SUD, PSTD and ED—disorders which have been recorded by some authors to meet the so-called definition of “internalising disorders”. These are conditions characterized by internal distress that include the DSM-5 [99] diagnoses of generalized anxiety disorder, social anxiety disorder, MDD, panic disorder and OCD, which are all thought to share the common trait of neuroticism. Neuroticism is defined as a genetically inherited trait that reflects individual differences in response to subjective distress and negative affectivity, but it is not itself considered to be a form of psychiatric disorder [100]. Nevertheless, all “internalizing disorders” involve dysfunctions in different neural systems and are differentially impacted by traumatic or chronically stressful events [101]. Interestingly, generalized anxiety disorder, social anxiety disorder, MDD, panic disorder and OCD are commonly treated with SSRIs, tricyclic antidepressants and different classes of drugs that target serotoninergic neurotransmission, thereby indicating a common neurobiological background.

To summarize, the advantages of using ketamine or esketamine can be recapitulated as the following: (i) no requirement for daily administration, as the molecules are administered for limited periods, thereby increasing a patient’s compliance and reducing the stigma of prolonged and daily intake of medications; (ii) the availability of nasal spray formulations that, in the case of esketamine, provide better handling and fewer safety profile issues and side effects; and (iii) in the case of concomitant medications, the possibility to reduce doses, thereby again improving safety profiles and reducing side effects [11,101].

### 4.5. Risk of Bias

**Figure 2:**
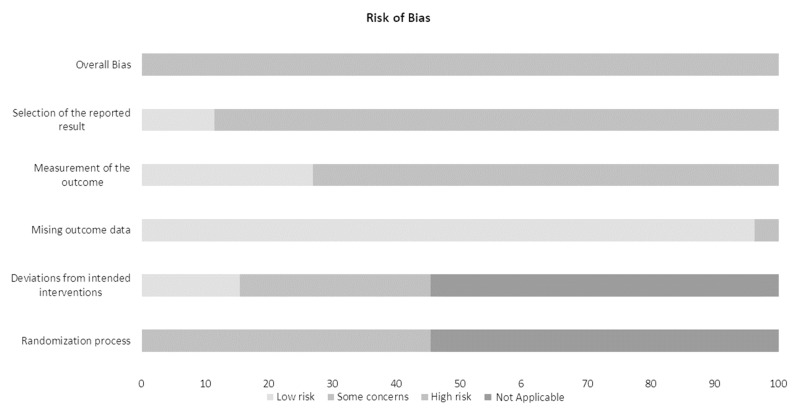
Risk of bias.

Considering the heterogeneity of the articles selected, the risk of bias was computed using RoB v.2.0 (Figure 2). 

Parameters “Mising outcome data” and “Selection of the reported results” were found to have a low risk or some concerns while, for selected parameters (e.g., “Randomization process” and “Deviation from intended process”), the assessment of risk of bias was not applicable.

### 4.6. Study Limitations

Despite the important data, the present review represents only a first assessment of data on the use of ketamine and esketamine and its effectiveness on OCD, ED and SUD. The first limitation of the present review was the predominance of heterogeneous studies, small samples and crossover designs. Comparative studies with conventional therapeutic strategies were not reported in the current literature. A second limitation was related to the use of a saline placebo and not of a real control for ketamine’s psychotomimetic effects. Moreover, studies had limited duration of follow-up; therefore, it was not possible to estimate the long-term benefit and potential long-term consequences of ketamine use. This point will require additional attention from researchers. Lastly, this review only included studies published in English.

## 5. Conclusions and Future Directions

For several reasons, it is not an easy task to establish a clear relation between glutamatergic dysfunction and OCD spectrum, or to understand this relationship’s exact functional meaning. Firstly, glutamatergic neurons are present in almost all brain circuitries and are therefore difficult to specifically localize a glutamatergic dysfunction in a specific brain area [5]. Secondly, while most studies converged on a hyper-glutamatergic state in OCD patients, it is noted that pro-glutamatergic agents also show some positive effects for OCD symptoms [69].

Many interesting new angles are offered by the implementation of ketamine and esketamine or, in general, psychedelic drugs for the treatment of many psychiatric and neurological conditions. These methods could be useful in those situations of non-response/resistance to standard pharmacological and psychological therapies that are currently observed in everyday clinical practice. Research efforts are needed to clarify the many unknowns related to the effects of these substances. These uncharted waters include short- and long-lasting neurobiological changes, impacts on the modulation of network activity and the functional connectivity of the brain, as well as the effects of drug-driven structural and functional modulations on mind function. However, a safe assumption is that joint efforts employing psychedelic-assisted psychotherapy can provide much-needed therapeutic options for OCD, MDD, SUD, ED and more. This is a brave new world that we need to explore.

## Figures and Tables

**Table 1:** Main findings of retrieved studies.

	Sample (Mean Age/Age, SD)	Intervention	Control	Dosing	Duration	Population	Concomitant Drug	Outcome	Adverse Events Recorded
Case reports/case series									
**Adams et al., 2017 [40]**	1 M (20 years-old)	CBT therapy was accompanied for weeks 3–6 by twice-weekly administration of racemic ketamine hydrochloride (50 mg). He returned home 2 weeks after the final ketamine treatment and continued twice-weekly CBT for one month and once-weekly CBT for another month	NA	IN ketamine, 10 mg: five 10 mg doses (5 mg/nostril) over a 20-minute period using an intranasal atomizer	16 weeks	He had a principal diagnosis of OCD, comorbid major-depressive disorder with chronic suicidal ideation, social anxiety disorder, and a history of bulimia nervosa, refractory to several pharmacological treatments	NA	Rapid and drastic reductions in suicidal ideation following the first week of ketamine treatment; overall findings included a 3-point reduction in YBOCS; and improved compliance with ERP	IN ketamine was well tolerated. Psychotomimetic effects were mild and passed within one hour of drug administration
**Lalanne et al., 2016 [41]**	1 F (36 years old)	To assist her with her opioid withdrawal, she received ketamine and two days after ketamine initiation her opioid treatment was gradually reduced (10% reduction in the initial dosage each two days)	NA	1 mg/kg ketamine oral solution	Two weeks	She was admitted to the rheumatology department of Strasbourg University Hospital suffering from lumbar pain with hyperalgesia and addiction to painkillers	NA	The patient dramatically reduced the dosage of opioid painkillers and ketamine was withdrawn without any withdrawal symptoms	Not recorded
**Mills et al., 1998 [42]**	15 F (33.3 years)	Rationale: since ketamine prevents all new memories being established, it would not be appropriate to use continuous ketamine treatment, so intermittent infusions were used to prevent further stimulation of the NMDA receptors which are essential for long-term potentiation to be enhanced, breaking the cycle of recall—re-stimulation. Using nalmefene, it was hoped to prevent loss of consciousness during the ketamine infusion	NA	Infusion of 20 mg per hour ketamine for 10 h	14 months	Patients with an ED (bulimia/anorexia) in a chronic refractory state	Amitriptyline; nalmefene 20 mg twice daily	Nine responders showed prolonged remission when treated with two to nine ketamine infusions at intervals of 5 days to 3 weeks, which persisted long after discharge from hospital. This was shown by a return to normal eating behaviour and the acceptance of normal increase in weight. They found it easier to maintain social contact, and discussed plans their future. Clinical response was associated with a significant decrease in the Compulsion score	Transient hallucinations, headache, nausea,
**Ocker et al., 2020 [43]**	1 M (55 years old)	5-day continuous ketamine infusion in combination with CBT to successfully taper 330 mg of daily morphine equivalents off	NA	-day 1: a continuous IV ketamine infusion was started at 10 mg/h (0.09 mg/kg/h) along with a clonidine 0.3 mg patch and lorazepam 0.5 mg every 6 h. -day 2: ketamine infusion was titrated up in 10 mg/h (0.09 mg/kg/h) increments, with these titrations occurring in the morning and in the afternoon; -day 3: ketamine infusion was titrated from 35 mg/h (0.3 mg/kg/h) to 50 mg/h (0.43 mg/kg/h); -day 4: ketamine up to a maximum rate of 70 mg/h (0.6 mg/kg/h); -day 5: ketamine infusion was reduced by 50% and then discontinued in the afternoon	5 days	Patient with complex regional pain syndrome	-day 1: hydromorphone IV was also provided, together with oral acetaminophen, ibuprofen, and pregabalin; -day 3: oxycodone 5 mg every 4 h as needed was then started for withdrawal symptoms	After discharge he continued his treatment course of CBT every 3 to 4 weeks as an outpatient via telemedicine. He did not use any of the prescribed opioids after discharge. The patient was administered one additional 5-day infusion at 6 months and remained opioid-free while experiencing a major improvement in function and lifestyle that he still maintains	On day 3 he experienced hallucinations, for which 2 doses of lorazepam were given. Other adverse effects: nausea, vomiting, anxiety, blurry vision
**Omoigui et al., 2011 [44]**	Case 1: F (42 years old); Case 2: M (20 years old)	Subanaesthetic doses of IV ketamine in adults have been used as a bridge to treat opiate withdrawal in the induction phase of buprenorphine	NA	Case 1: a Normal Saline infusion was initiated and the patient was given Ketamine 5 mg IV bolus (repeated once), magnesium sulfate 3.5 g IV infusion, and ketorolac 60 mg IV bolus to treat her symptoms. She was also started on the Suboxone induction dose of 2–0.5 mg film—2 films two times a day for the first day, then 2 films four times a day for the second day and thereafter. Case 2: he was treated with suboxone for heroin dependence, without complete resolution of abstinence. Suboxone dosage was increased to 4 tablet (2 mg each)/3 times day. Then, a normal saline infusion was initiated and he was given ketamine 5 mg IV bolus (repeated once) and 60 mg of ketorolac IV bolus	NA	Case 1: history of polysubstance Abuse (cocaine, marijuana, alcohol, hydromorphone, methadone, topiramate, and celecoxib, as well as psychiatric drugs such as aripiprazole, trazodone, and bupropion), bipolar disorder, anorexia nervosa, and schizophrenia; Case 2: substance abuser (heroin, marijuana, tobacco)	Cases 1–2: suboxone	Case 1: Six days later, in the follow-up visit, the patient reported having been free of withdrawal symptoms for 18 h post IV treatment. Case 2: patient reported complete resolution of the abdominal pain, and that his headache was reduced to a 1 out of 10. A follow up after two months since his first visit to the clinic revealed that patient was completely withdrawal free thus accomplishing another successful shift to buprenorphine	Not recorded
**Rodriguez et al., 2011 [45]**	F (24-year-old)	She received two double-blind IV infusions over 40 min given 1 week apart of saline or 0.5 mg/kg ketamine hydrochloride	Saline	40-minute IV ketamine infusion	One week	Patient with treatment-resistant OCD	NA	A minimal reduction in obsessions during the first infusion (placebo/saline) have been reported), and a complete cessation of obsessions during the second infusion (ketamine). Obsessions partially re-emerged 40 to 230 min post-infusion, plateauing until post-infusion Day 2; the obsessions did not return to baseline levels until post-infusion Day 7. Ketamine might have rapid anti-obsessional effects that persist from 1 to 7 days post-infusion, long after the drug has cleared	During ketamine infusion she reported lightheadedness, dry mouth, and feelings of unreality (CADSS = 19) that resolved 5 min after the infusion stopped
**Schwartz et al., 2021 [46]**	4 F (36.75 years)	Ketamine IM (dosing 0.5–0.80 mg/kg) administered, with repeat dosing at 4–6 weeks intervals	NA	IM ketamine administration, except for the first 2–3 doses administered IV for cases 3 and 4. The standard dosing of 0.5 mg/kg was the initial dose. Subsequent doses were titrated to response, side effects, and safety. An additional injection of 0.3–0.4 mg/kg to alternate deltoid 24 h after the first dose was administered if previous dose of 0.5 mg/kg was partially effective/ ineffective	Up to 12 months	Patients had been chronically ill with an ED for more than 7-years duration; subjects met current criteria for TRD without psychotic features	Case 1: aripiprazole 10 mg/die and fluoxetine 40 mg/die; Case 2: escitalopram 20 mg/die; Case 3: sertraline 200 mg/die, aripiprazole 10 mg/die; case 4: venlafaxine XR 150 mg/die, lamotrigine 200 mg/die, and topiramate	Clinically meaningful changes in depression and to a lesser degree anxiety and ED symptoms	Dissociative effects lasting 30–90 min, post-treatment sleepiness, and occasional mild headache
**Sharma et al., 2020 [47]**	14 (7M, 7F; 36.2 years, 12.9)	Repeated ketamine infusions	NA	Infusion of ketamine at 0.5 mg/kg over 40 min (number of infusions ranged from 2 to 10)	Up to three weeks	Adults diagnosed with SRI-resistant OCD	SRIs	Statistically significant reduction in OCD illness severity following ketamine. However, only three patients (21%) showed a clinical response (one remission, and two partial response)	Not recorded
**Scolnick et al., 2020 [48]**	29 years old F	Ketogenic diet used specifically for the treatment of anorexia nervosa, followed by a short series of titrated IV ketamine	NA	Infusion of ketamine at 0.5 mg/kg	6 weeks	Woman who struggled with severe and enduring anorexia nervosa for 15 years	Not recorded	Complete remission of severe and enduring anorexia nervosa, with weight restoration, and sustained cessation of cognitive and behavioral symptoms, for 6 months.	Not recorded
**Veraart et al., 2020 [13]**	1 F (55 years old)	Oral esketamine treatment	NA	Oral esketamine treatment started at 0.5 mg/kg twice weekly and was titrated to 2.0 mg/kg	6 weeks	MDD who previously was resistant to ECT and DBS comorbid with psychotic and obsessive symptoms	Venlafaxine 300 mg; Clozapine 450 mg; Glycopyrronium 0.7 mg; Movicolon daily; Nitrazepam 5 mg three times per week. DBS settings were kept stable at 3.0 V, pulse width 60 and frequency 180 Hz	Decrease in the IDS-SR score from 54 to 30 and in the HDRS score from 24 to 6. The patient reported an overall good response and started to function again in important domains of life. Both her auditory hallucinations and obsessive- compulsive symptoms decreased. She currently continues esketamine treatment twice weekly at home, and has been in remission for 18 months	Apart from temporary dizziness, no adverse events occurred
**Randomised-controlled trials (RCT)**									
**Dakwar et al., 2017 [49]**	20 (48.6 years, 6.1)	Participants were hospitalized up to 3 times in a controlled research unit for 6 days at a time, and each hospitalization was separated by two weeks. Each 6-day hospitalization involved an initial 2-day washout period; in the second and third hospitalizations, participants were randomized (1:1) for ketamine or midazolam under double-blind conditions	In addition to the sham infusion in phase 1 (saline over 52 min), on day 4 of phases 2 and 3 a 52-min infusion of 0.025 mg/kg midazolam was administered as active control	52-min subanesthetic infusions of ketamine (0.71 mg/kg)	60 days	Non-depressed, cocaine dependent individuals disinterested in treatment or abstinence. Exclusion criteria were: (i) physiological dependence on opioids, alcohol, benzodiazepines; (ii) history of psychotic or dissociative symptoms; (iii) current depressive or anxiety symptoms; (iv) a first-degree family history of psychosis; (v) obesity (BMI > 35); (vi) cardiovascular/pulmonary disease	Administration of two cocaine doses (25 mg cocaine base) starting at 1 p.m. on day 3 of each hospitalization, with each dose separated by 15 min; and on Day 5, participants underwent a session of five choices (25 mg cocaine or $11, every 15 min), starting at around 2 p.m.	Ketamine, as compared to the control, significantly decreased cocaine self-administration by 67% relative to baseline at greater than 24 h post-infusion	Acute dissociation that resolved within 30 min post-infusion. No other adverse effects
**Dakwar et al., 2018 [50]**	20 (49.8 years, 5.7)	Participants were hospitalized up to 3 times (for 6 days at a time), and each hospitalization was separated by two weeks. Each 6-day hospitalization involved an initial 2-day washout period; in the second and third hospitalizations, participants were randomized (1:1) for ketamine or midazolam under double-blind conditions	In addition to the sham infusion in phase 1 (saline over 52 min), on day 4 of phases 2 and 3 a 52-min infusion of 0.025 mg/kg midazolam was administered as active control	52-min subanesthetic infusions of ketamine (0.71 mg/kg)	60 days	Nontreatment seeking cocaine dependent individuals. Exclusion criteria were: (i) physiological dependence on opioids, alcohol, benzodiazepines; (ii) history of psychotic or dissociative symptoms; (iii) current depressive or anxiety symptoms; (iv) a first-degree family history of psychosis; (v) obesity (BMI > 35); (vi) cardiovascular/ pulmonary disease	Administration of two cocaine doses (25 mg cocaine base) starting at 1 p.m. on day 3 of eachhospitalization, with each dose separated by 15 min; and on Day 5, participants underwent a session of five choices (25 mg cocaine or $11, every 15 min), starting at around 2 p.m.	Improvements in cocaine self-administration, cocaine use, and cocaine craving were found to be mediated by HMS score, suggesting that mystical-type phenomena played a central role in the behavioural impact of ketamine	Not recorded
**Dakwar et al., 2019 [52]**	55 (14F, 41M) cocaine-dependent adults (47.0 years; 9.3)	Patients were randomly assigned to receive an IV infusion of ketamine or midazolam during a 5-day inpatient stay, during which they also initiated a 5-week course of mindfulness-based relapse prevention	IV midazolam	40-minute IV infusion of ketamine (0.5 mg/kg)	2 weeks	Cocaine-dependent individuals of the NewYork State Psychiatric Institute clinical research unit	NR	48.2% of individuals in the ketamine group maintained abstinence over the last 2 weeks of the trial, compared with 10.7% in the midazolam group. The ketamine group was 53% less likely to relapse (dropout or use cocaine) compared with the midazolam group, and craving scores were 58.1% lower in the ketamine group throughout the trial; both differences were statistically significant.	Infusions were well tolerated, and no participants were removed from the study as a result of adverse events
**Dakwar et al., 2020 [51]**	40 (53 years, 9.8)	Participants were randomly assigned Ketamine (N = 17) or the active control (N = 23) during the second week of a 5-week outpatient regimen of motivational enhancement therapy	Midazolam (0.025 mg/kg)	52-minute IV administration of ketamine (0.71 mg/kg)	5-week	Treatment-seeking adults with alcohol dependence	NR	Ketamine significantly increased the likelihood of abstinence, delayed the time to relapse, and reduced the likelihood of heavy drinking days compared with midazolam	Infusions were well tolerated, with no participants removed from the study as a result of adverse events
**Das et al., 2020 [53]**	90 (35F, 55M; 27.5 years, 8.1)	Ketamine infusion followed retrieval of alcohol-maladaptive rewarding memories (RET + KET) or control reward memories (No RET + KET). A third group retrieved alcohol- maladaptive rewarding memories prior to IV placebo (RET + PBO)	Saline solution	IV ketamine (0.5 mg/kg) for ten days	9 months	Hazardous/harmful drinking patterns, recruited via open internet advertisements. Inclusion criteria were: scoring > 8 on the AUDIT; not meeting SCID criteria for AUD at screening; consuming > 40 (M) or > 30 (F) units/week (1 unit = 8 g ethanol), primarily drinking beer; non-treatment seeking	NA	Ketamine infusion produced a reduction in the reinforcing effects of alcohol among harmful drinkers. A rapid and lasting reduction in number of drinking days per week and volume of alcohol consumed was observed, with no rebound to baseline for at least 9 months following manipulation. Control groups receiving retrieval or ketamine alone did not show such changes in reward-related responses to alcohol, although the latter group did show some reduction in drinking	Not recorded
**Rothberg et al., 2020 [54]**	40 (21F, 19M) alcohol-dependent (53.0 years, 9.8)	52-minute infusion of ketamine 0.5 mg/kg or midazolam, which they received on a designated quit-day during the second week of a five-week motivational enhancement therapy regimen; alcohol use was monitored for the subsequent 3 weeks at each twice-weekly visit	IV midazolam	52-minute infusion of ketamine 0.5 mg/kg	5-week trial	Alcohol-dependent individuals with a minimum use criteria of at least 4 days of heavy drinking (>4 drinks/day for M; >3 drinks/day for F) over the past 7 days, or minimum weekly use of 35 drinks for M and 28 for F. Exclusion criteria: (i) dependence on another substance; (ii) active depressive disorder, or past/current bipolar or other psychotic disorders	NA	Ketamine led to significant reduction in at-risk drinking. Mystical-type effects (by HMS) were found to mediate the effect of ketamine on drinking behaviour	Not recorded
**Open-label studies**									
**Bloch et al., 2012 [55]; Niciu et al., 2013 [56]**	10 (7F, 3M; 37.3 years)	Subjects were hospitalized for 1 week prior to and 1 week following ketamine infusion in order to maintain a consistent environment in which to assess OCD symptoms. Structured clinical ratings were performed at screening/baseline, 1, 2, 3 h and 1, 2, 3, 5 and 7 days following ketamine infusion (YBOCS; HDRS; CADSS; CGI)	NA	40-minute single IV infusion of 0.5 mg/kg of ketamine	One week	Subjects with treatment-refractory OCD, seven of whom had active comorbid depression	NA	Both OCD and depression symptoms demonstrated a statistically significant improvement in the first 3 days following infusion compared to baseline, but the OCD response was <12%. Also, although ketamine had no sustained anti-obsessive effect (no significant sustained reduction in YBOCS), four of the seven subjects with comorbid depression experienced an acute antidepressant effect. However, we unexpectedly observed delayed-onset dysphoria, worsening anxiety and suicidal thinking in two of the three subjects with OCD and extensive psychiatric comorbidity but minimal depressive symptoms at the start of infusion	Transient increase in systolic blood pressure (<30% above baseline, max 160/80). Half of subjects reported some dissociative symptoms during infusion, but CADSS scores remained low. Subjects reported gaps in memory (n = 3), sensory distortions (i.e. perioral paresthesia, n = 2), feeling that time was moving in slow motion (n = 2), disconnection from reality (n = 1)
**Jovaiša et al, 2006 [57]**	58 (7F, 51M; 23.6 years, 3.05)	Opiate-dependent patients were enrolled in a randomized, placebo-controlled, double-blind study. Patients underwent rapid opiate antagonist induction under general anesthesia. Prior to opiate antagonist induction patients were given either placebo or subanesthetic ketamine infusion	Saline	Infusion of 0.5 mg/kg/h ketamine	4 months	Opiate-dependent patients (duration of substance was abuse more than one year), aged 18–35 years, without other minor comorbidities. Exclusion criteria: (i) patients with a current history of long-acting opiate or polysubstance abuse; (ii) acute medical or surgical condition; (iii) pregnancy	Not recorded	Ketamine group presented better control of withdrawal symptoms, which lasted beyond ketamine infusion itself. Subanesthetic ketamine infusion was an effective adjuvant in the correction of acute precipitated opiate withdrawal. Significant differences between ketamine and control groups were noted in anesthetic and early postanesthetic phases. There were no long-term effects on treatment of opiate dependence after 4 months. Data supported the hypothesis that NMDA antagonists may selectively interfere with expression of opiate withdrawal	Not recorded
**Krupitsky et al., 2007 [58]**	59 (10F, 49M; 22.6 years ± 3.9)	Study of the efficacy of single versus repeated sessions of KPT in promoting abstinence in people with heroin dependence	IM ketamine (2.0 mg/kg)	Patients received a KPT session prior to their discharge from an addiction treatment hospital, and were then randomized into two treatment groups. Participants in the first group received two addiction counseling sessions followed by two KPT sessions, with sessions scheduled on a monthly interval (multiple KPT group). Participants in the second group received two addiction counseling sessions on a monthly interval, but no additional ketamine therapy sessions (single KPT group)	14 months	Detoxified inpatients with heroin dependence. Exclusion criteria: (i) ICD- 10/DSM-IV criteria for a psychotic/mood disorder; or alcoholism or polydrug dependency; (ii) advanced neurological, cardiovascular, renal, or hepatic diseases; (iii) pregnancy; (iv) family history of psychiatric disorders listed above; (v) cognitive impairment; (vi) active tuberculosis or current febrile illness; (vii) AIDS; (viii) significant laboratory abnormality; ix) pending legal charges with potential incarceration; x) participation in other treatment/study or concurrent treatment in another substance abuse program	Not recorded	At one-year follow-up, survival analysis demonstrated a significantly higher rate of abstinence in the multiple KPT group. Thirteen out of 26 subjects (50%) in the multiple KPT group remained abstinent. compared to 6 out of 27 subjects (22.2%) in the single KPT group (*p* < 0.05).	The only side effect noted in all participants was an acute increase in systolic and particularly diastolic blood pressure of 20% to 30% during the ketamine psychotherapy session
**Pradhan and Rossi, 2020 [59]**	3 (NA)	Single IV ketamine administration over a 45-minute period. A one-week washout period followed prior to the start of rTMS, performed for five sessions (10 Hz and 3000 stimulation pulses applied to the right DLPFC) over one-two weeks. Five sessions of TIMBER-based therapy were carried out during the same two weeks period that rTMS was performed. Home practice was then carried out two times daily with additional sessions on an as-needed basis for craving	NA	Infusion of 0.75 mg/kg weight-based dose capped at 745 mg total	2 weeks	All patients were diagnosed with OUD and completed three months of residential treatment prior to treatment with ketamine infusion	NA	Combination therapy with ketamine, rTMS and TIMBER is feasible in patients with OUD and reduces craving, promotes abstinence, and reduces the amount used in patients with OUD	Not recorded
**Rodriguez et al., 2013 [60]**	15 (7F, 8M; 34)	Patients received two 40-min IV infusions, one of saline and one of ketamine, spaced at least 1-week apart.	Saline	IV infusion of 0.5 mg/kg of ketamine	2 weeks	Drug-free OCD adults with near constantobsessions (YBOCS)	Not recorded	Ketamine’s effects within the crossover design showed significant carryover effects (ie, lasting longer than 1 week). Specifically, those receiving ketamine reported significant improvement in obsessions (measured by OCD-VAS) during the infusion compared with subjects receiving placebo. One-week post-infusion, 50% of those receiving ketamine met criteria for treatment response (≥35% YBOCS reduction) vs 0% of those receiving placebo. Rapid anti-OCD effects from a single IV dose of ketamine can persist for at least 1 week in some OCD patients with constant intrusive thoughts. Glutamate neurotransmission can reduce OCD symptoms without the presence of an SRI and is consistent with a glutamatergic hypothesis of OCD	Not recorded
**Rodriguez et al, 2015 [63]**	16 (7F, 9M, 32.9 years, 7.5)	Patients received two IV infusions at least 1 week apart, one of saline and one of ketamine, while lying supine in a MR scanner. The order of each infusion pair was randomized. Levels of GABA and Glx were measured in the MPFC before, during, and after each infusion	NA	IV infusion of 0.5 mg/kg of ketamine	NA	OCD with at least moderate symptoms (YBOCS score ≥16).	Not recorded	No change in Glx; a significant increase in GABA/W following ketamine infusion	Not recorded
**Rodriguez et al., 2016a [64]**	12 (4F, 8M; 33.6 years)	Open-label memantine was started at 5 mg daily and titrated by 5 mg weekly to 10 mg twice daily for up to 6 weeks. Memantine was continued to 12 weeks in those with treatment response either to ketamine (≥35% YBOCS reduction one week after IV ketamine) or current response to memantine (≥35% YBOCS reduction from pre- to post- 6 weeks of memantine)	NA	IV infusion of 0.5 mg/kg of ketamine	12 weeks	OCD with at least moderate symptoms (YBOCS score ≥16). At the time of the memantine trial, all were unmedicated and 2 had symptoms of mild to moderate depression	Not recorded	No significant response to 12 weeks of memantine post ketamine (YBOCS)	Not recorded
**Rodriguez et al., 2016b [61]**	10 (aged 18–55 years)	In an open-label design, participants received a single 40-minute IV infusion of ketamine (dose = 0.5 mg/kg), followed by 10 one-hour exposure sessions delivered over two weeks	NA	40 min IV infusion of 0.5 mg/kg of ketamine	Two weeks	OCD outpatients with YBOCS score ≥ 16	Not recorded	Significant reduction in OCD severity over 2 weeks of ERP	Not recorded
**Rodriguez et al., 2017 [62]**	Of the 23 adults (18–55 years) with OCD who contacted the clinic, 2 participants finally completed the study, a 36-years-old man, and 20-years-old woman. Patient who was randomized to midazolam was offered open-label intranasal ketamine 50 mg after study completion	Single nasal administration	IN midazolam 4 mg	IN ketamine, 50 mg	Two weeks	OCD outpatients with YBOCS score ≥ 16, and on stable psychotropic medication for at least 6 weeks prior to enrollment. Exclusion criteria included severe depression or comorbid psychiatric or medical conditions	Not recorded	Neither patient met OCD treatment response criteria	Poorly tolerated: dissociation (e.g. body feeling unusually large, colors seemed brighter than expected, and time slowed) that lasted for 45 min after administration; nausea and headache that resolved 110 min post-administration
**Yoon et al., 2019 [65]**	5 (1F, 4M; 49.2 years)	Patients received injectable naltrexone (380 mg once 2–6 days prior to the first ketamine infusion) and repeated IV ketamine treatment once a week for 4 weeks (a total of 4 ketamine infusions)	NA	IV ketamine treatment (0.5 mg/kg once a week for 4 weeks	8 weeks (2 phases: a 4-week ketamine treatment and a 4-week follow-up phase)	Patients with current major depressive disorder and alcohol use disorder	Naltrexone	The combination of naltrexone and ketamine was associated with reduced depressive symptoms. Also, 80% (4 of 5) of patients reported improvement in alcohol craving and consumption as measured by the Obsessive Compulsive Drinking Scale	The combination treatment was safe and well tolerated in all participants. No serious adverse effects were reported in the trial
**Double-blind trial**									
**Dakwar et al., 2014a [66]**	8 M (47.7 years, 5.6)	Three 5- minute IV iinfusions were administered in a randomized, double-blind manner (ketamine or lorazepam). Infusions were separated by 48 h, and assessments occurred at baseline and at 24 h post-infusion	IV lorazepam 2 mg	IV ketamine(0.41 mg/kg or 0.71 mg/kg)	10 days with a 4-weeks follow up	Eight volunteers with active DSM-IV cocaine dependence not seeking treatment or abstinence	Not recorded	Glutamatergic actions of sub-anesthetic ketamine extend beyond anti-depressant efficacy and may also address dependence-related adaptations, demonstrating promising effects on motivation to quit cocaine and on cue-induced craving, 24 h post-infusion	Not recorded
**Dakwar et al., 2014b [67]**	8 M (18–55 years)	Three 52-min infusions separated by 48 h were administered in a randomized double-blind manner to each participant ketamine 0.41 mg/kg or 0.71 mg/kg or lorazepam 2 mg (LZP). There were three possible orders: K1, K2, LZP; LZP, K1, K2; and K1, LZP, K2. K1 always preceded K2 for safety reasons. Participants were interviewed 20 min and 1 h post-infusion, with special attention given to persistent symptomatology that might merit concern, such as sedation, psychosis or dissociation.	IV lorazepam 2 mg	IV ketamine 0.41 mg/kg (K1) or 0.71 mg/kg (K2)	4 weeks	Cocaine dependent individuals not seeking treatment or abstinence, actively using freebase (“crack”) cocaine, and describing a history of cue-induced craving	Not recorded	All psychoactive effects, including dissociative and mystical-type phenomena, resolved within 20 min postinfusion. Both doses of ketamine led to significant elevations in HMS score relative to lorazepam. Additionally, K2 led to significantly greater mystical-type effects by HMS compared to K1. HMS score, but not CADSS score, was found to mediate the effect of ketamine on motivation to quit cocaine 24 h postinfusion.	Not recorded
**Krupitsky et al., 2002 [68]**	70 (15F, 55M; 22.3 years, 2.7)	Patients were randomly assigned to one of two groups receiving KPT involving two different doses of ketamine. Both the psychotherapist and patient were blind to the dose of ketamine. The therapy included preparation for the ketamine session, the ketamine session itself, and the post session psychotherapy	NA	IM ketamine 2.0 mg/kg versus 0.2 mg/kg	5 days	Seventy detoxified heroin-addicted patients	Not recorded	The results of this double blind randomized clinical trial of KPT for heroin addiction showed that high dose (2.0 mg/kg) KPT elicits a full psychedelic experience in heroin addicts as assessed quantitatively by the Hallucinogen Rating Scale. On the other hand, low dose KPT (0.2 mg/kg) elicits ‘‘sub-psychedelic’’ experiences and functions as ketamine-facilitated guided imagery. High dose KPT produced a significantly greater rate of abstinence in heroin addicts within the first two years of follow-up, a greater and longer-lasting reduction in craving for heroin, as well as greater positive change in nonverbal unconscious emotional attitudes than did low dose KPT	Not recorded

AUDIT: Alcohol Use Disorders Identification Test; CADSS: Clinician-Administered Dissociative States Scale; CBT: cognitive behavioural therapy; CGI: Clinical Global Impression; DB: double-blind; DBS: deep brain stimulation; DLPFC: dorsolateral pre-frontal cortex; DSM: Diagnostic Statistical Manual; ECT: electroconvulsive therapy; ED: eating disorder; ERP: ERP—exposure and response prevention; F: female; GABA: gamma amino butyric acid; Glx—glutamate + glutamine; HDRS: Hamilton Depression Rating Scale; HMS: Hood Mysticism Scale; IDS-SR: Inventory of Depressive Symptomatology Self-Report; IV: intravenous; KPT: ketamine-assisted psychotherapy; M: male; MDD: major depression disorder; MR: magnetic resonance; NA: not applicable; OCD: obsessive-compulsive disorder; OUD: opioid use disorder; RCT: randomised-controlled trial; rTMS: repetitive transcranial magnetic stimulation; SCID: Structured Clinical Interview for DSM; SD: standard deviation; SRI: serotonin reuptake inhibitors; TIMBER: Trauma Interventions using Mindfulness Based Extinction and Reconsolidation of memories; TRD: treatment-resistant depression; VAS—visual analog scale; XR: extended-release; YBOCS: Yale-Brown Obsessive-Compulsive Scale.

**Table 2:** Use ok ketamine and esketamine as a treatment for the obsessive-compulsive disorder (OCD), substance use disorders (SUD) and eating disorders (ED).

	Mechanism of Action Mediated by Ketamine/Esketamine	Therapeutic Regimen	Current Evidence	Ref.
**OCD**	Esketamine/ketamine decreases NMDA activity which is thought to be related to OCD compulsive behaviours Esketamine/ketamine activates synaptic plasticity and functioning Increased signaling via mTOR, BDNF and GSK-3 pathways	Single or repeated doses (2 to 10) of IV Ketamine 0.5 mg/kg IN Ketamine 50 mg single dose/10 mg in five doses Oral S-ketamine treatment started at 0.5 mg/kg twice weekly and was titrated to 2.0 mg/kg continuing for 18 months S-ketamine treatment twice weekly	Ketamine significantly improves obsessive-compulsive symptomatology. Effects are rapid (occurring in hours to minutes) but short-lasting (days to weeks); concomitant CBT prolongs ketamine effects A maintenance treatment with oral S-ketamine combined with experimental deep brain stimulation showed long-term (18 months) beneficial effects	[1,2,3,4,13,26,55,61,62,63,64]
**SUD**	Improved prefrontal cortex glutamate homeostasis causing synaptic improvements Improved aberrant changes in synaptic and structural plasticity following repeated drug exposure	IV Ketamine (as a single or repeated) infusion of 0.50 mg/kg weight-based dose, administered over a 45-minute period Repeated administration of IV ketamine (0.41 and 0.71 mg/kg) IM ketamine (0.2 mg/kg vs 2.0 mg/kg) Nalltrexone (380 mg once administered 2–6 days prior to the first ketamine infusion) and repeated IV ketamine treatment (0.5 mg/kg once a week for 4 weeks)	Combining ketamine, repetitive transcranial magnetic stimulation (rTMS) and mindful-ness therapy appeared effective for maintenance of abstinence in OUD, particularly reducing craving Repeated administration of IV ketamine effectively enhanced the motivation to quit and reduced cocaine craving High dose IM Ketamine (2.0 mg/kg) in conjunction with psychotherapy produced a significantly greater rate of abstinence in heroin addicts, a greater and longer-lasting reduction in craving for heroin, as well as greater positive change in nonverbal unconscious emotional attitudes than did low dose Ketamine (0.2 mg/kg IV ketamine administered prior to rapid opiate antagonist induction showed that ketamine could suppress physiologic response to opiate withdrawal The combination of injectable naltrexone and IV ketamine during an 8-week open-label pilot study was effective in reducing depressive symptoms in alcohol addicts	[26,49,50,51,52,57,59,65,70,71,72]
**Eating Disorders**	Esketamine/ketamine antagonism on NMDA receptors of hippocampus whose activation is thought to be related to recall of anorexic thoughts Action on BDNF, GABA, and NMDA glutamate receptors Restoring glutamatergic neurotransmission, which has been implicated in key AN symptoms including set shifting, interoceptive awareness, and compulsive behaviours	Short series of titrated IV ketamine at dose of 0.75 mg/kg infused over 45 min. (four infusions during 14 days, at doses titrated to 1.0 mg/kg, 1.1 mg/kg, and 1.2 mg/kg)	IV ketamine led to complete remission of severe and enduring AN, with weight restoration, and sustained cessation of cognitive and behavioural symptoms, for 6 months	[42,49,87,88,89]
